# Choosing between Orthotopic Neobladder and Ileal Conduit after Radical Cystectomy: Tools for Assessing Patient-Specific Characteristics and Enhancing the Decision-Making Process—A Review of Current Studies

**DOI:** 10.3390/jcm13123506

**Published:** 2024-06-15

**Authors:** Maciej Trzciniecki, Paweł Kowal, Jan Kołodziej, Tomasz Szydełko, Anna Kołodziej

**Affiliations:** 1 Department of Urology, Regional Specialist Hospital, 51-124 Wroclaw, Poland; 2 Medical University, 50-367 Wroclaw, Poland; 3 Clinical Department of Minimally Invasive and Robotic Urology, Wroclaw Medical University, 50-367 Wroclaw, Poland

**Keywords:** radical cystectomy, urinary diversion, ileal conduit, neobladder, cognitive impairment, frailty, dexterity, patient counseling, bladder cancer, urologic oncology, supportive care

## Abstract

**Objective**: The aim of the study was to find tools to assess patient characteristics that would help in choosing between orthotopic neobladder and ileal conduit in patients undergoing radical cystectomy. An additional goal was to search for aids that improve preoperative counseling to support patients in the decision-making process. **Methods**: A systematic review of MEDLINE, Web of Science, and Scopus databases was conducted, according to the Preferred Reporting Items for Systematic Reviews and Meta-analyses (PRISMA) statement, in April 2024. Inclusion criteria were specified in PICO format. Two reviewers independently screened titles/abstracts and full papers. Upon study selection, the results and conclusions from the studies were abstracted and quantitatively summarized in the results section of this article. **Results**: Seven articles, involving a total 834 patients, were included. One article described frailty, two reviewed cognitive status, one article described functional dexterity, one described personality, two articles reviewed patients’ values and goals, and one article reviewed role of patient–physician dialogue in the context of choosing UD after RC. The reviewed articles identified tools and approaches that could be valuable in evaluating the suitability for continent urinary diversion (CUD) or incontinent urinary diversion (ICUD). **Conclusions**: This is the first systematic review that summarizes the new available methods of patient assessment which improve preoperative counseling and choosing the most suitable UD after RC. Efficient tools for this purpose are still missing, and further studies that will aid in creating a simple aid for patient selection are necessary.

## 1. Introduction

Radical cystectomy (RC) is a standard treatment of patients with muscle invasive bladder cancer. Ileal conduit (IC) or orthotopic neobladder (ONB) are most used urinary diversions (UDs) [1]. Contraindications for ONB are well known and are listed in the Methods Section. Nevertheless, most patients are still suitable for both types of UD [2]. Health-related quality of life (HRQoL) after RC has been compared in many studies, without giving clear advantages to any type of UD [3,4]. The limitations of current studies make characterization of the superiority of one type of UD impossible [3]. Therefore, the choice of UD should be individualized [3,4], and a pre-operative counseling, with patients preferences incorporated, is advised, however, unfortunately, clear answers as to how this should be performed have not yet been provided in the literature [2]. 

The appropriate choice of urinary diversion is essential because there are differences between continent urinary diversion (CUD) and incontinent urinary diversion (ICUD) in term of postoperative complications rates. Short- and intermediate-term complications rates in radical cystectomy are similar, however long-term complications seem more frequent in ONB compared to IC [5]. The popularity of robot-assisted radical cystectomy has been growing in recent years, interestingly, with increasing number of performed CUDs, which in some centers has become the UD of choice [6,7]. The first article comparing ONB and IC in robot-assisted radical cystectomy showed that type of UD was significantly related to postoperative complications, with higher complication rates, and longer operative time and length of hospital stay in the ONB group [7]. Importantly, despite differing complication rates, the type of urinary UD does not affect mortality, which is consoling information for urologists and patients during preoperative counseling [8].

Finally, making the right choice between CUD and ICUD is essential because of the different level of difficulty in handling both UD types and the different types of complications. ICUD, such as IC, is a simple urinary diversion that provides a constant urine drainage and, except for stoma hygiene, does not require special attention from the patient, thus complications are rare, but, in return, the patient must accept a stoma. CUD, such as ONB, provides a natural body image and almost, physiological urethral urination, which is important for many patients. Due to the collection of urine in a non-physiological intestinal reservoir, this type of UD is more difficult to manage and carries a risk of higher-grade complications. ONB does not give the sensation of urinary urgency, so the patient must be disciplined to urinate at regular time intervals, also during the night, to prevent bladder overfilling and secondary vesicoureteral reflux, followed by metabolic acidosis or chronic kidney injury. In addition, patients must be able to learn how to properly empty the ONB by abdominal pressing with simultaneous relaxation of the urethral sphincter and, if stricture of the urethrovesical anastomosis occurs, they must be manually skillful enough to be able to perform chronic self-catheterization. Lack of efficient bladder emptying, retention, and vesico-ureteric reflux can lead to serious health consequences, including chronic kidney damage, metabolic acidosis, osteoporosis, recurrent urinary tract infections, or urolithiasis. Therefore, the ability to preoperatively assess whether a certain patient will be able to efficiently handle ONB or, if not, whether it would be better to use a simple UD, such as IC, seems to be an important problem, a solution to which would help avoid many complications accompanying incompetent CUD handling.

Assessment of cognitive impairment (CI), frailty, dexterity, or factors related to patient preferences are recommended [9], however, correlation of these factors is usually evaluated with postoperative outcomes, not upon qualification for UD [10,11,12]. 

The aim of this study was to perform a systematic review of literature in search of tools facilitating the selection between CUD and ICUD and improving preoperative counseling, mainly for supporting patients in the decision-making process. To our knowledge, this is first systematic review of literature focusing on this topic. 

## 2. Methods 

### 2.1. Eligibility Criteria

The inclusion criteria were specified in PICO format and are presented in Table 1. 

Exclusion criteria were as follows: full text not available, full text in language other than English, case reports, non-original data such as reviews, commentaries, and editorials. Publications related to patient selection for continent urinary diversion based on general contraindications. 

General contraindications according to latest EAU guidelines on muscle-invasive and metastatic bladder cancer for the year 2024 include: debilitating psychiatric and neurological diseases, severe liver or renal impairment, urothelial carcinoma positive margins, short life expectancy, extensive bladder cancer to the prostatic urethra in men, or bladder neck in women. Other contraindications include urethral stricture, preoperative radiotherapy, and impaired or damaged rhabdosphincter.

### 2.2. Information Sources

A systematic search of MEDLINE, Web of Science, and Scopus databases was conducted independently by two authors on 18 April 2024.

### 2.3. Search Strategy

The electronic search strategy included the following search query: (clinical AND decision AND making) OR psychological OR cognition OR cognitive OR neuropsychological OR (patient AND selection) OR (clinical AND test) OR dexterity OR “mini-mental” OR “clock-drawing” OR frailty OR choice OR “preoperative counseling”) AND (“urinary diversion” OR “ileal conduit” OR neobladder OR cystectomy).

### 2.4. Selection Process

Following the search strategy, screening of titles and abstracts was performed independently by two reviewers. Studies were excluded if they did not meet the criteria for eligibility. A consensus on research included for evaluation was achieved through discussion between the reviewers based on the criteria of eligibility. 

### 2.5. Data Collection Process 

Upon study selection, results and conclusions from the studies were abstracted and summarized in the Results Section of this article. 

### 2.6. Registration

The study is not registered on the PROSPERO database.

## 3. Results

### 3.1. Study Selection

**Figure 1:**
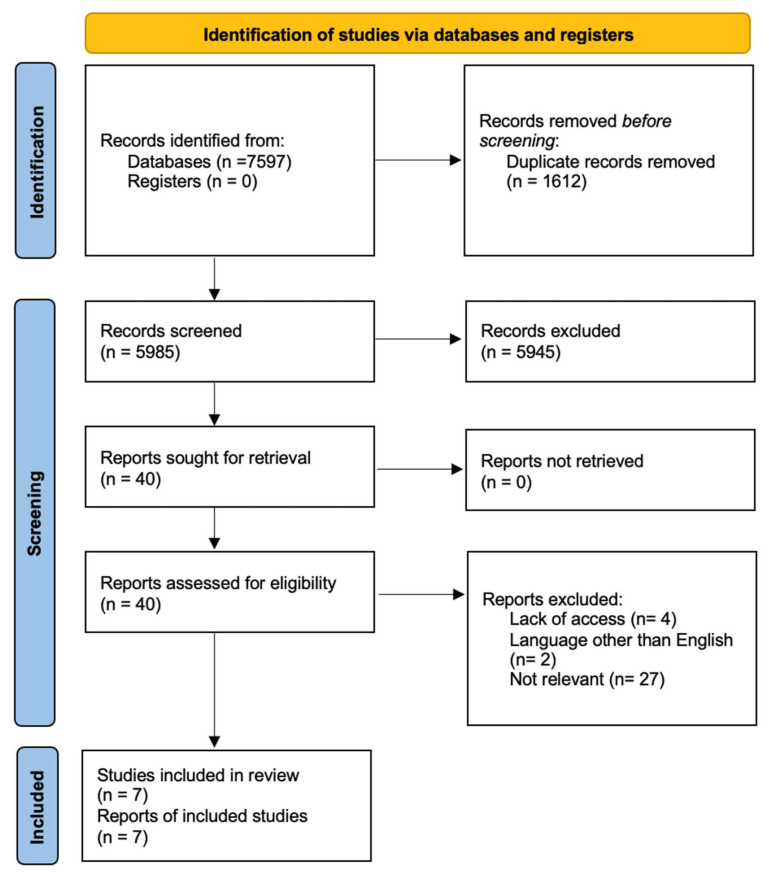
Search strategy according to the PRISMA protocol.

We found 7597 records (2233 in MEDLINE, 3036 in Web of Science, and 2328 in Scopus), and 1612 duplicates were removed. All titles with an abstract were reviewed and 40 qualified for a full review of eligibility. Seven articles met the inclusion criteria and were qualitatively summarized in this review. The described selection process is shown in the PRISMA flowchart (Figure 1).

### 3.2. Study Characteristics

Considering the seven studies that met inclusion criteria, a total of 834 patients with bladder cancer treated with RC were included. The characteristics of the included studies are shown in Table 2.

### 3.3. Results of Individual Studies

Study 1. Design. Prospective observational study form Japan which described the relationship between patient frailty and type of UD [13]. Patients. Study involved 88 patients, with median age of 68 years, undergoing RC. Methods. Frailty was assessed with the frailty discriminant score (FDS), Fried phenotype criteria (FP), and modified frailty index (mFI). Next, patients were classified into the ONB (*n* = 54) or non-ONB (*n* = 34) group based on tumor status, patient comorbidities, performance status and patient’s preference. Surgeon was blinded to frailty tests results. Authors compared frailty with UD type, postoperative complications, and overall survival. Results. There were no significant differences between preoperative status between ONB and non-ONB groups. There was a significant difference in frailty between groups for the FDS (*p* = 0.018) and FP (*p* = 0.001). Higher FDS and FP test results were significantly associated with the choice of non-ONB. In addition, mFI and FDS were correlated with higher postoperative complication rates and poor overall survival, respectively. Conclusion: Frailty assessment might support decision-making in choosing UD in patients undergoing RC.

Study 2. Design. Combined retrospective and prospective observational study from Germany aimed to assess the influence of cognitive status and functional dexterity on HRQoL, functional outcomes, and medical care situation in patients undergoing RC with UD [14]. Patients. A total of 106 (*n* = 77 assessed retrospectively, *n* = 29 assessed prospectively) patients with CUD (*n* = 29 neobladders, *n* = 51 ileocecal pouches) and ICUD (*n* = 26 IC) were involved in the study, with a median age of 66 years. Methods. Patients from the prospective group were preoperatively assessed for CI with the Mini-Mental Status test (MMS)/clock drawing test, and for functional dexterity with the functional dexterity test (FDS). Next, they filled out questionnaires referring to HRQoL, sexuality, and functional parameters related to types of UD, which was repeated 3 and 6 months after surgery. The retrospective group was assessed only once. Patients were divided into three groups according to the type of UD. Results. Older age was significantly correlated with lower MMS test results (*p* = 0.04) and lower FDS results (*p* = 0.02). Reduced dexterity was also significantly correlated with lower MMS test scores (*p* = 0.01). Patients with IC had worse dexterity (median FDS 33.8 vs. 26.6 and 26.7 s, *p* = 0.01) and were significantly older (77 vs. 66 years and 62 years, *p* ≤ 0.01) than patients with CUD (neobladders and ileocecal pouch, respectively). Any or moderate constraints related to UD occurred significantly more often in patients with reduced dexterity, compared to patients with correct dexterity (52.6% vs. 32.3%, respectively, *p* ≤ 0.01), and similarly, in cases of severe constraints (28.9% vs. 14.7%, respectively, *p* ≤ 0.01). Patients with reduced MMS test results significantly more often had moderate (41.9% vs. 30.1%, *p* = 0.02) and severe constraints (23.3 vs. 14.3%, *p* = 0.03) related to UD. Conclusions: Assessment of functional dexterity and cognitive status might be beneficial while choosing type of UD.

Study 3. Design. Prospective observational study from Germany described cognitive function in patients undergoing RC [15]. Patients. A total of 51 patients (median age was 69 years, 80% of patients were male) undergoing RC were involved. Methods. Patients were prospectively examined with three tests for CI (DemTect—Dementia Detection Test, MMSE—Mini-Mental State Examination, and clock drawing test) two days prior to RC. The admitting surgeon, blinded for cognitive test results, evaluated patients by ranking them from 1 to 10 points, with >5 accepted as the cut-off for ONB. Next, authors correlated the frequency of mild CI, the evaluation of the attending physician, and perioperative complications. Results. A total of 29 patients received IC, 16 patients received ONB, and six patients received other UD. Of the patients, 35% had CI. Patients with mild CI in DemTect significantly more often developed high grade adverse events according to the Clavien Dindo classification (in dichotomous and continuous analysis, *p* = 0.042 and *p* = 0.0238, respectively). Life-threatening complications occurred in four (29%) out of 14 patients with mild CI in DemTect, compared to two (5%) out of 37 patients with a normal test result. It is mportant to note that DemTect results were only weakly correlated (*rs* = 0.149, *p* = 0.424) with physicians’ subjective assessment of the patients’ suitability for ONB. The admitting doctor assessed 20 patients as suitable for ONB, while 5/20 (25%) patients had a pathological DemTect result. Conclusion: CI is common among patients undergoing RC. Evaluation of CI might help in predicting surgical complications and planning surgical approach in this group of patients.

Study 4. Design. Prospective, multicenter observational study from Germany described the role of personality and anxiety in patient preference in the context of choosing UD [16]. Patients. The authors recruited 180 patients (mean age 68.77 years, 75% patients were male) waiting for consultation on RC and selection of UD. Methods. Before consultation, patients were asked to fill questionnaires assessing personality (BFI-10), anxiety (STAI), participation preference in shared-decision making (API and API-uro), and treatment preferences referring to UD. Results. Most of the patients (78.9%) reported clear treatment choice, with 44.4% and 34.4% selecting CUD and ICUD, respectively. Of the patients, 32.2% had significant anxiety symptoms. Most of the patients preferred to transfer decision-making to healthcare professionals. Patients choosing CUD had significantly higher (*p* < 0.01) results in conscientiousness than patients choosing ICUD. Higher preference of participation and autonomy in uro-oncological decision-making processes was corelated with higher neuroticism (*p* < 0.05) and anxiety (*p* < 0.05). Conclusion. Consideration of personality aspects and anxiety level might enhance shared decision-making processes.

Study 5. Design. A retrospective survey study from the United Kingdom analyzed patients’ values and worries in relation to the choice of UD after RC [17]. Patients. The sample included 62 patients after RC, with either IC (14 men, 24 women, mean age 62), or ONB (18 men, 6 women, mean age 52). Methods. Patients filled out three questionnaires evaluating quality of life (EORTC QLQ-30), the Life Values Inventory (LVI), and the bladder reconstruction satisfaction questionnaire (BRSQ). The BRSQ referred to patients’ opinions on the aspects of reconstruction that were most crucial to them in making their decision related to UD. All patients had chosen their UD, and only “very satisfied” and “satisfied” patients (*n* = 55) were analyzed. Results. Cancer removal and returning to normality were most important goals for patients before RC, regardless of the type of UD. Patients with IC found maintenance, (*p* < 0.001) and severity of surgery, (*p* < 0.001) more important. Patients with ONB found the ability to return to normality more important compared to those opting for IC (*p* < 0.001) Conclusion. There are some values and concerns that affect patients’ choice of UD, which could support shared decision-making while choosing UD following RC. 

Study 6. Design. In a retrospective survey study from the USA, authors created a scale for assessing patients’ goals in relation to different types of UD [18]. Patients. A total of 215 patients (mean age 69 years, 80% patients were male) who underwent RC with either IC (*n* = 167) or ONB (*n* = 48) reconstruction were involved in the study. Methods. Using interviews with patients and formative focus groups, the author identified six and four factors related with goals that could be achieved with IC and ONB, respectively. IC-related goals included items as follows: “having the least risky procedure because of my age”, “having the shortest possible operation”, “having the least risky procedure because of other health problems”, “avoiding relearning how to empty my bladder”, “higher recovery speed”, and “avoidance of catheter use”. ONB items included: “avoid having a urostomy bag”, “being close and intimate with my partner without having a bag”, “having my body function as naturally as possible”, and “continuing my active lifestyle”. Authors chose patients who were preoperatively eligible for both types of UD and mailed them a survey with questionnaire including adapted goal alignment and dissonance questions. Presence of goals specific to the type of UD was correlated with goal alignment in those who received that diversion, and goal dissonance in those who did not. Results. Most value goals among patients with ONB were “avoid having a urostomy bag” and “be close and intimate with my partner without having a bag”, while ONB dissonant goals were neutral in this group. ONB patients described a strong desire to maintain the integrity of their own body and function. Patients with IC had neutral values for all isolated goals. Conclusion. Presented dissonance scale may facilitate patients’ decision-making process referring to choosing the right UD.

Study 7. Design. A prospective study from the USA described the impact of patient–physician dialogue in selecting UD [19]. Patients. The study included 132 patients undergoing RC. Methods. Two groups were formed: patients under 70 years old were in group I (*n* = 69), and patients over 70 years old in group II (*n* = 63). Patients were evaluated for their eligibility for either ONB and IC, or for IC only, based on cancer characteristics and comorbidities assessment. Results. A total of 73 ICs and 59 ONBs were performed. In the absence of contraindications, patients were offered to choose UD. If so, 85% of patients from group I, and 55% patients from group II chose ONB (*p* < 0.05). IC as the only option was offered to 16% and 65% of patients from group I and II, respectively. Despite the suggestion, six patients asked for ONB, which was performed as requested. Despite developing postoperative complications, all six patients were satisfied with their decision. Conclusion. Most patients submit to physicians’ suggestions related to UD, but when patients are allowed to choose, younger patients prefer ONB, while older patients tend to choose IC.

### 3.4. Synthesis of Results

Studies were conducted in Japan (1), Germany (3), United Kingdom (1), and USA (2). None of the studies were randomized. Four studies were prospective, two retrospective, and one was combined. The period of studies ranged from 2003 to 2021. One article described frailty, two reviewed cognitive status, one article described functional dexterity, one described personality, two articles reviewed patients’ values and goals, and one article reviewed role of patient–physician dialogue in the context of choosing UD after RC. We divided seven presented studies into two main groups. 

The first group included three studies comparing measurable patient health-related characteristics, including frailty, cognitive status, and functional dexterity. These conditions may affect the patient’s ability to cope with a more complex type of UD. In the first prospective study, Okita et. al demonstrated a correlation between frailty and choice of non-ONB, and higher postoperative complications and poor overall survival [13]. However, some drawbacks, such as small sample size, short observational period, and decision-making for ONB selection by only one surgeon’s included selection bias, need to be highlighted. Two studies analyzed cognitive status impairment. Kalagirou et al. used the MMS and clock drawing test, and found correlation between all results and postoperative stoma constraints [14]. Same correlation was found also with stoma constraints and reduced dexterity. Small samples, partially the retrospective character of the study, and non-validated questionnaires for UD-related constraints are minor points of this study. In contrast, Grunewald et al. used DemTect and MMSE/clock drawing test, and found correlation with DemTect results but not for MMSE/clock drawing test results in postoperative complication, according to the Clavien Dindo classification [15]. Limitations again were small sample size, and short period of observation. 

In the second group, we found four studies focusing on shared decision-making and a more personalized approach to the patient while choosing UD, involving studies describing personality [16], patients’ values and goals [17,18], and role of patient–physician dialogue in the context of choosing UD [19]. The main drawbacks of all four studies are small sample sizes, but most of all, the correlations between all assessed features and their impact on choice of UD were not evaluated. Assessing patient personality and preferences in the context of choosing a UD seems to be helpful in selecting patients that need more assistance in decision-making, but without giving any guidance for physicians on which UD to suggest [16]. This is an especially important issue, as in one study the authors demonstrated that more than 80% of patients accept physician advice related to UD [19]. Two articles presented questionnaires (ten-item decision dissonance scale and BRSQ) assessing patient goals and the aspects of reconstruction that were crucial to them in making their decision related to UD, however, questionnaires were not validated and designed for study purposes [17,18]. Furthermore, questionnaires were filled out postoperatively in both studies, therefore risk of post-hoc justification related to the choice of UD exists. 

## 4. Discussion

Most of the patients undergoing RC were eligible for either CUD or ICUD. The lack of tools that would facilitate advising the most suitable UD for certain patients may lead to the wrong therapeutic decisions. More accurate patient selection based on validated tests would be a useful tool for the clinician, and for patients, to provide better functional outcomes, but such tools are lacking [14]. In this review, we investigated the impact of few different factors on this process.

Okita et al. found correlation between increased frailty and choosing non-orthotopic UD, higher postoperative complications, and poor overall survival [13]. What seems very important is that when created by the admitting surgeon, ONB and non-ONB groups were comparable according to ECOG PS and comorbidities. However, frailty was significantly different between both groups, which indicates the importance of frailty assessment with dedicated tools, because this cannot be detected on standard preoperative evaluation, and frailty itself have impact on postoperative complications and survival. However, assessing frailty requires either a special device for handgrip strength measurement, or extensive analysis of the medical history, which seems complicated for clinical use. Therefore, verifying usefulness of an easier test, such as a simplified frailty measurement tool, in this application in further studies might be beneficial [11,20,21]. 

Impaired cognitive function is strongly associated with higher rates of severe perioperative complications and occurrence of postoperative constraints associated with the handling of UD [14,15]. Analogous to the assessment of frailty, when cognitive function is not evaluated preoperatively, some patients with CI may be assessed as eligible for CUD, which initially is associated with higher complications rates than ICUD [15]. Thus, routine assessment of CI may reduce the number of misqualifications for CUD and later complications. In two mentioned articles, authors evaluated the MMS, clock drawing test, and DemTect scores, but assessment with two different endpoints makes choosing one go-to test for cognitive assessment impossible at this moment.

When ONB in considered, it is important to take into account that up to 25% of patients develop hypercontinence, and some require clean intermittent catheterization (CIC) [22,23]. Reduced dexterity is associated with increased postoperative constraints with CIC [14]. Higher BMI, age, non-vaginal/ non-nerve sparing RC and ONB reconstruction are risk factors for requiring CIC postoperatively [22]. Evaluation of dexterity and ability to assess the likelihood of requiring CIC would help the selection of patients suitable for CUD.

Sufficient information of patients whose preferences are considered is associated with lower rates of decision regret related to UD [24]. Patients mostly submit to the physician’s suggestion, which shows how important and responsible a role they have in preoperative counseling [19]. Therefore, the ability to distinguish between patients who want to delegate decision-making to the doctors and those who want to participate in this process (e.g., by analyzing patients’ personality or anxiety level) is crucial [16]. Thus, shared decision-making processes in term of choosing the best possible UD for certain patients should be constantly developed in further studies.

In the two retrospective studies presented, authors developed scales and guidelines that facilitate the conscientious choice of UD [17,18]. Based on these facilitations, patients prior to RC can better identify themselves as suitable for a particular UD type, and make a more informed decision [17,18].

To summarise the reviewed articles, it seems that further studies on improving preoperative patient assessment should be focused on two main fields. The first one contains measurable characteristics of patients, such as frailty, cognitive status, and functional dexterity. Those conditions, which are not routinely assessed on preoperative counseling, have an impact on postoperative complications and seem to have influence on constraints related to urinary diversion. If further studies could compare available tools for assessing cognitive impairment, frailty, and dexterity before radical cystectomy, and then in follow-up checks, relationships between the results of these tests and some form of measurement of UD-related satisfaction level might provide answers for our needs. The second field incorporates patient-tailored approaches. We must remember that ultimately, the patient decides the type of urinary diversion, therefore the second important area of research should focus on creation of a tool which, after analysis of features like personality, patients’ values, and goals, allows the selection of the most suitable UD for a certain patient.

Finally it seems worth recalling that CUD correlates with a higher number of postoperative adverse events, including UD-related complications [5]. As is well known, CUD is more complicated to handle compared to IUD. If the patient is unable to cope efficiently with UD, this can be expected to generate UD-related complications. If an ideal tool were created to properly qualify patients for more complex forms of UD, would the number of postoperative adverse events in the CUD group decrease? This is one of the questions that brought us to write this article. If so, it could prove that complication rates and HRQoL are rather dependent on the precision of preoperative counseling for UD type, rather than on the type itself. The current trend of increasing rates of ONB is probably due to improved surgical techniques, rather than more accurate qualification, which may increase complications rates and have a negative impact on HRQoL in this group of patients if our theory is true.

Our aim was to review the literature in search for aids for improving this process. Surprisingly we found that there is a huge gap between the need for and the availability of tools for decision-making. However, we found few promising studies evaluating this topic from different perspectives. We hope that our review will be a good summary of the state of knowledge and an inspiring starting point for further studies on this still poorly understood subject.

In our study, we did not analyze sexual quality of life in terms of choice of UD, because the quality of sexual life after RC is poor regardless of chosen method and is thus not the main goal of the operation [4]. However, sexual sparring techniques are reserved for highly motivated patients and can be used equally in both CUD and ICUD. All specific RC-specific HRQoL questionnaires contain sexual activity-related questions, and in relation to latest systematic reviews, the determination of which UD is better in this context is impossible at this moment, especially since most patients undergo non-sexual-preserving surgeries [3,25].

The limitations of this systematic review include, first and foremost, the small number of studies meeting the inclusion criteria, a low amount of high-level evidence and well-designed trials, and the large heterogeneity of topics that make formulating concise conclusions impossible. Several studies were single center, had a small sample size, and formulated conclusions based on non-standardized self-designed questionnaires, and others were only retrospective or had no or short follow-up.

## 5. Conclusions

This is the first systematic review that summarizes the new available methods of patient assessment that could improve preoperative counseling and choice of the most suitable UD after RC. Our most important conclusion is that such tools are missing. We have searched three databases and from initial 7597 articles, we found only seven articles meeting the inclusion criteria. Surprisingly, all studies present vastly different approaches to the same topic of the choice of UD, which made comparing the studies and the effectiveness of the presented tools impossible. Frailty, cognitive status, functional dexterity, personality, anxiety, patient values, and preferences were investigated in terms of their usefulness in choosing the UD. The results of the presented research do not yet provide ready clinical aids, but they are a cornerstone and thus set a direction for further research, which seems necessary in the light of the presented data.

## Figures and Tables

**Table 1:** PICO.

Patients	Patients with Bladder Cancer Undergoing RC with CUD or ICUD
Interventions	Assessment of patients’ psychological status OR assessment of cognitive function OR assessment of dexterity OR assessment of frailty OR assessment of preferences OR assessment of anxiety OR assessment of personality OR assessment of goals and values OR assessment of patient–physician dialogue
Comparison	None or standard counseling
Outcomes	Choice of either CUD or ICUD

**Table 2:** Summary of characteristics of included studies.

Author Year	Design Country	Number of Patients	Age, Median (Years)	CUD	ICUD	Measurement Tools	Results	Advantages of Measurement Tools	Disadvantages of Measurement Tools
1. Okita et al. [13] 2020	Prospective observational study, Japan	88	68	54	34	Frailty discriminant score (FDS), Fried phenotype criteria (FP), modified frailty index (mFI)	Higher FDS and FP test scores were significantly associated with choice of ICUD. Frailty was correlated with higher postoperative complication rates and poorer overall survival	FDS, FP, and mFI are widely used, validated, objective, easy to perform	FDS and FP need special tools for grip strength measurement
2. Kalagirou et al. [14] 2019	Combined retrospective and prospective observational study, Germany	106	66	80	26	Mini-Mental Status test (MMS)/clock drawing test, Functional Dexterity Test (FDS). Questionnaires related to HRQoL, sexual and functional parameters related to type of UD	Older age was significantly correlated with lower MMS test results and lower FDS results. Patients with reduced MMS test results and patients with reduced dexterity had constraints related to UD significantly more often	FDS, MMS, and clock drawing test are widely used, validated, objective, easy to perform	Functional Dexterity Test needs special equipment (wooden insertion plate), questionnaires related to HRQoL, sexual and functional parameters related to type of UD are not validated and were developed by authors for the study purpose, and questionnaires are a subjective assessment made by patients
3. Grunewald et al. [15] 2022	Prospective observational study, Germany	51	69	16	35	Dementia Detection Test (DemTect), Mini-Mental State Examination, (MMSE), clock drawing test	Patients with mild CI in DemTect significantly more often developed a high grade adverse event, according to the Clavien Dindo classification. DemTect results were only weakly correlated with physicians’ subjective assessment of the patients’ suitability for ONB.	DemTect, MMSE, and the clock drawing test are widely used, validated, objective, and easy to perform correlation of tests results with adverse events classified with widely used Clavien Dindo scale	Test results correlated also with physicians’ subjective assessment of the patients’ suitability for ONB
4. Köther et al. [16] 2022	Prospective, multicenter observational study, Germany	180	68.77	-	-	Questionnaires assessing personality (BFI- 10), anxiety (STAI), participation preference in shared decision-making (API and API-uro), and treatment preferences referring to UD	Most of the patients (78.9%) reported clear treatment choice and preferred to transfer decision-making to healthcare professionals. Patients choosing CUD had significantly higher results in conscientiousness than patients choosing ICUD. Higher preference of participation and autonomy in uro-oncological decision-making processes was correlated with higher neuroticism	Validated questionnaires, easy to apply in clinical practice	results are based on self-report tests; subjective assessment
5. Reed and Osborne [17] 2019	Retrospective survey study, United Kingdom	62	62	24	38	Questionnaires evaluating quality of life (EORTC, QLQ-30), the Life Values Inventory (LVI), and the bladder reconstruction satisfaction questionnaire (BRSQ)	Cancer removal and returning to normality were the most important goals for patients before RC, regardless of the type of UD. Patients with IC found maintenance and severity of surgery important, and ability to return to normality was important to patients with ONB.	BRSQ—tool enhancing more personalized approach to the patient in decision-making related to UD	BRSQ is not validated tool created for the study purpose. Patient goals were evaluated postoperatively—risk of post-hoc justification of choice
6. Leo et al. [18] 2019	Retrospective survey study, USA	215	69	48	167	Questionnaire included adapted goal alignment and dissonance questions	Most value goals among patients with ONB were “avoid having a urostomy bag” and “be close and intimate with my partner, without having a bag”, while ONB dissonant goals were neutral in this group. ONB patients described a strong desire to maintaining integrity of their own body and function. Patients with IC had neutral values for all isolated goals	Ten-item decision dissonance scale—tool enhancing a more personalized approach to the patient in decision-making related to UD	Ten-item decision dissonance scale is not a validated tool, created for study purpose Patient goals evaluated postoperatively—risk of post hoc justification of choice
7. Katkoori et al. [19] 2010	Prospective study, USA	132	Group I < 70 years old, Group II > 70 years old	59	73	Evaluation for UD type based on cancer characteristic and comorbidity assessment; in the absence of contraindications, patients were offered the choice of UD	Younger patients preferred ONB, while older patients tended to choose IC; 85% of patients from group I, and 55% patients form group II have chosen ONB. IC was offered as the only option to 16% and 65% of patients from Group I and II, respectively. Despite suggestion, six patients asked for ONB, which was performed as requested. Despite developing postoperative complications, all were satisfied with their decision.	Non-applicable (no specific tool or questionnaires used)	Non-applicable (no specific tool or questionnaires used)

## Abbreviations

IC—ileal conduit, ONB—orthotopic neobladder, CUD—continent urinary diversion, ICUD—incontinent urinary diversion, UD—urinary diversion, RC—radical cystectomy, CIC—clean intermittent catheterization, HRQoL—health-related quality of life, CI—cognitive impairment.
